# Sequential Use of Recombinant Porcine Factor VIII and Early Emicizumab in Acquired Hemophilia A: A Case Series

**DOI:** 10.1002/jha2.70335

**Published:** 2026-07-02

**Authors:** Yusuke Yamada, Takashi Onaka, Kazunori Imada

**Affiliations:** ^1^ Department of Hematology Japanese Red Cross Osaka Hospital Osaka Japan

**Keywords:** acquired hemophilia A, recombinant porcine FVIII, susoctocog alfa

## Abstract

**Background:**

Acquired hemophilia A (AHA) is a rare autoimmune bleeding disorder requiring prompt hemostatic control and sustained prevention of rebleeding. Recombinant porcine factor VIII (rpFVIII, susoctocog alfa) controls acute bleeding, while emicizumab provides prophylaxis. Real‐world data on their sequential use are limited.

**Objective:**

To describe clinical outcomes of three patients with AHA who received rpFVIII for initial hemostasis followed by early emicizumab.

**Methods:**

This case series describes three adults with AHA who received 200 U/kg rpFVIII for acute bleeding, early emicizumab loading on Days 2–3 followed by weekly maintenance, and individualized prednisolone. FVIII activity and inhibitor titers were measured using an emicizumab‐neutralized assay with anti‐idiotype antibodies.

**Results:**

Single‐dose rpFVIII achieved hemostasis within 24 h in all patients. No breakthrough bleeding, thrombosis, or adverse events occurred. Inhibitor titers decreased under immunosuppression. Two patients showed FVIII recovery by Day 49. One patient with persistently low FVIII activity remained stable on emicizumab. Hemostasis was achieved even in one patient with a baseline inhibitor titer of 105.8 BU/mL.

**Conclusions:**

Sequential rpFVIII followed by early emicizumab maintained bleeding control until inhibitor reduction and FVIII recovery occurred. This strategy was feasible and safe in these three patients. Larger studies are needed.

**Trial Registration:**

The authors have confirmed clinical trial registration is not needed for this submission.

## 1 Introduction

Acquired hemophilia A (AHA) is a life‐threatening bleeding disorder caused by autoantibodies that neutralize coagulation factor VIII (FVIII) [1]. Treatment aims are immediate hemostasis and the prevention of rebleeding by eradicating the inhibitor, and therapy selection requires balancing hemostatic efficacy against thrombotic risk. International recommendations endorse recombinant activated factor VII (rFVIIa), activated prothrombin complex concentrate (aPCC), or recombinant porcine FVIII (rpFVIII) for acute control, and existing data show no clear efficacy or safety advantage among these options [1]. Moreover, rpFVIII enables measurable FVIII replacement and typically achieves control within 24 h [2]. Despite its proven effectiveness in acute settings, repeated dosing may elicit anti‐porcine inhibitors that reduce its clinical response [3]. Emicizumab, a bispecific FVIII‐mimetic antibody, provides durable prophylaxis in AHA and reaches hemostatic levels within several days after initiation [4, 5]. Since it cannot control acute bleeding immediately, a practical approach is to combine agents for rapid control and then maintain bleeding prevention with emicizumab while immunosuppression clears the inhibitor. A previous case report described the salvage use of emicizumab after recurrent bleeding following initial treatment with rpFVIII [6]; however, data on sequencing rpFVIII with emicizumab remain limited. We herein report the cases of three patients managed with early emicizumab after rpFVIII‐induced hemostasis and describe their subsequent clinical courses.

## 2 Methods

We retrospectively reviewed the cases of three adults with AHA who were treated at our institution in 2025. The treatment sequence was selected by the treating physicians for each patient. All patients received susoctocog alfa at 200 U/kg for acute hemostasis. After hemostasis was achieved, emicizumab was initiated on Day 2 at 6 mg/kg and on Day 3 at 3 mg/kg, followed by 1.5 mg/kg weekly from Day 9. Prednisolone at 1 mg/kg was started on Day 1 and tapered individually.

FVIII activity was measured weekly with a one‐stage clotting assay, and inhibitor titers were assessed by the Bethesda assay. Samples obtained during emicizumab prophylaxis were neutralized with anti‐emicizumab idiotype antibodies before testing.

Recorded outcomes were time to hemostasis, FVIII recovery, inhibitor clearance, and adverse events. All patients provided written informed consent, and the study was approved by the institutional review board in accordance with the Declaration of Helsinki.

## 3 Results

**FIGURE 1:**
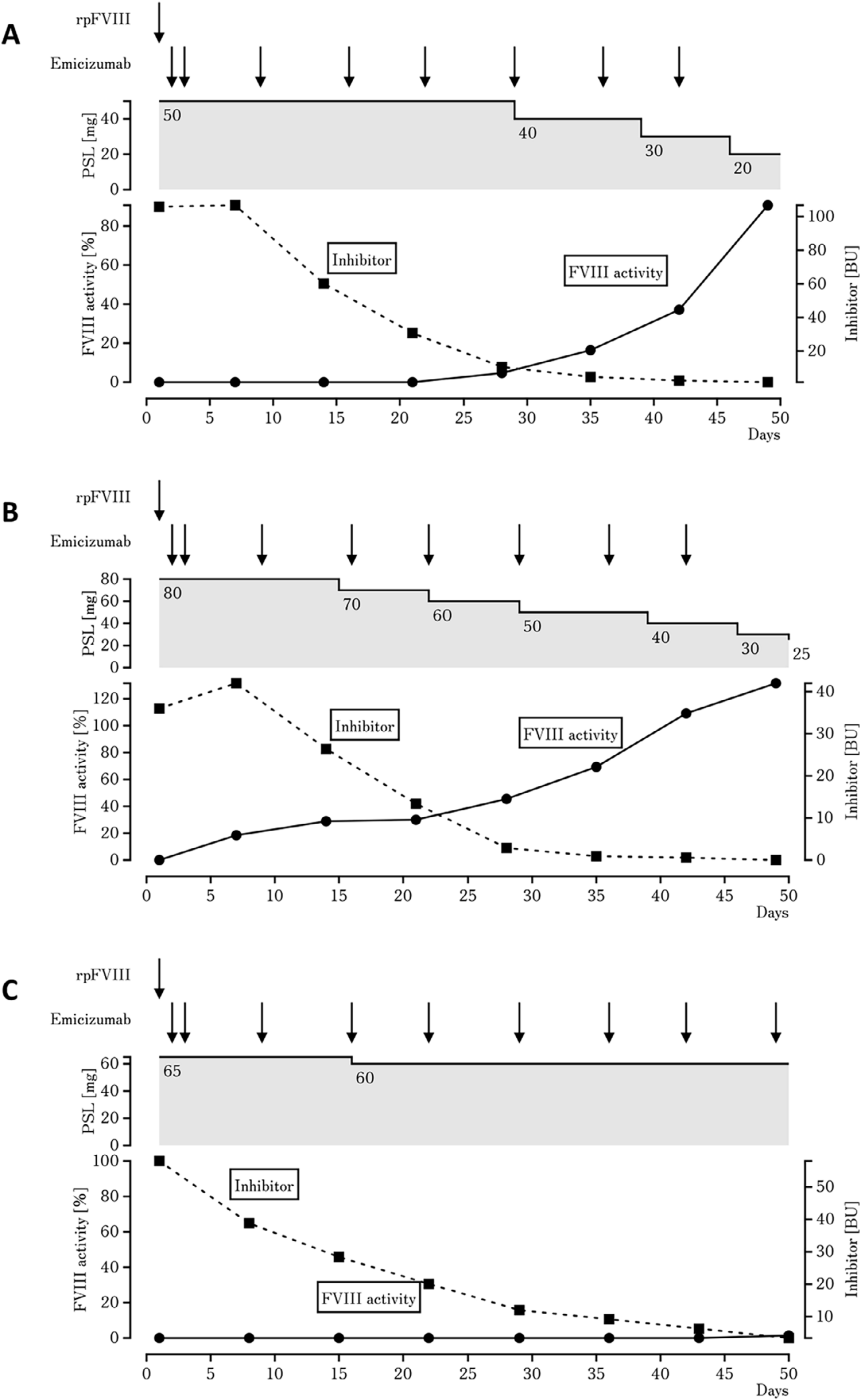
Early emicizumab initiation after a single effective rpFVIII infusion achieved hemostasis in three adults with acquired hemophilia A despite varying baseline inhibitor levels and FVIII recovery patterns during corticosteroid therapy. (A) Case 1, spontaneous intramuscular bleeding with a high inhibitor titer (105.8 BU/mL); hemostasis and FVIII recovery occurred after a single rpFVIII infusion and early emicizumab. (B) Case 2, postoperative intramuscular bleeding (FVIII < 1%, inhibitor 36.0 BU/mL); the patient achieved an early clinical and immunological response with corticosteroids. (C) Case 3, trauma‐associated subcutaneous bleeding; the patient showed delayed FVIII recovery despite clinical stability. By Day 49, FVIII activity recovered in Cases 1–2, whereas Case 3 showed delayed FVIII recovery. Upper panels show treatment timelines (arrows: rpFVIII and emicizumab dosing; gray bars: prednisolone). Lower panels show FVIII activity (%) and inhibitor titers (BU) over time. Inhibitor titers progressively decreased during corticosteroid therapy in all patients. BU, Bethesda unit; FVIII, factor VIII; PSL, prednisolone; rpFVIII, recombinant porcine factor VIII.

Figure 1 shows the clinical courses of three adults with AHA, including treatment timelines, FVIII activity, and inhibitor titers.

### 3.1 Case 1

A 79‐year‐old man with hypertension presented with spontaneous bilateral thigh hematomas. He had no known malignancy at presentation, although colorectal cancer was later diagnosed. On admission, his hemoglobin was 3.0 g/dL and aPTT was 150 s. He received red blood cell transfusions before being transferred to our hospital for further care. At our institution, his FVIII activity was < 1%, and an inhibitor titer of 105.8 BU/mL was observed. On Day 1, susoctocog alfa (rpFVIII) at 10,000 U and prednisolone at 50 mg were started. Within 24 h, his bleeding symptoms improved. Thigh pain and swelling decreased, and the worsening of his anemia stopped, indicating clinical hemostasis. Emicizumab was initiated on Day 2 (6 mg/kg) and Day 3 (3 mg/kg), followed by 1.5 mg/kg weekly. Inhibitor titers declined from 106.7 BU/mL (Day 7) to 30.7, 10.6, 4.7, and 1.5 BU/mL on Days 20, 27, 34, and 49, respectively, while his FVIII activity increased from < 1% to 4.7% (Day 27), 16.4% (Day 34), and 90.6% (Day 49). No breakthrough bleeding or thrombosis occurred (Figure 1A).

### 3.2 Case 2

A 59‐year‐old man with nonalcoholic steatohepatitis developed refractory postoperative intramuscular bleeding after femoral head arthroplasty. He had no malignancy, infection, or anticoagulant use. Initial tests showed FVIII activity < 1% and an inhibitor titer of 36.0 BU/mL. On Day 1, he received susoctocog alfa at 16,000 U and prednisolone at 80 mg, with bleeding controlled within 24 h. Emicizumab was initiated on Day 2 (6 mg/kg), followed by 3 mg/kg on Day 3 and 1.5 mg/kg weekly from Day 9. His FVIII activity increased to 45.5% (Day 28), 109.1% (Day 42), and 135.6% (Day 49), while his inhibitor titer fell to 2.9 BU/mL (Day 28) and was undetectable by Day 49. No breakthrough bleeding, thrombosis, or other adverse events occurred (Figure 1B).

### 3.3 Case 3

A 78‐year‐old woman with IgA bullous dermatosis was taking prednisolone at 8 mg daily. She fell at home and presented 2 days later with fatigue and extensive subcutaneous hematomas. Her hemoglobin was 4.8 g/dL and her anemia progressed despite transfusions. Initially, her FVIII activity was < 1% and her inhibitor titer was 58.0 BU/mL. On Day 1, prednisolone at 65 mg and susoctocog alfa at 12,000 U were given. Emicizumab was started on Day 2 with loading doses of 6 and 3 mg/kg on consecutive days, followed by 1.5 mg/kg weekly. Clinical hemostasis was confirmed the next day without further hematoma expansion or anemia progression. Inhibitor titers declined to 38.8, 28.4, 20.1, 12.0, 6.3, and 3.4 BU/mL on Days 8, 15, 21, 28, 42, and 50, respectively, whereas FVIII activity remained < 1% through most of the course and reached 1.4% by Day 50. There was no recurrence of bleeding or thrombotic events during the follow‐up period (Figure 1C).

### 3.4 Across‐Case Summary

All three patients received approximately 200 U/kg of rpFVIII as initial hemostatic therapy, achieved clinical hemostasis within 24 h, and then continued emicizumab without breakthrough bleeding or thrombosis. Inhibitor titers decreased under corticosteroid therapy in each case. FVIII activity recovered by Day 49 in two patients, whereas one patient showed delayed biochemical recovery despite clinical stability.

## 4 Discussion

This case series suggests that a single dose of approximately 200 U/kg of rpFVIII followed by early emicizumab achieves rapid and durable bleeding control during inhibitor reduction in AHA. While rpFVIII provides measurable FVIII replacement and achieves early, effective hemostasis in acute settings, emicizumab reaches protective levels within several days and prevents rebleeding.

In this series, all patients achieved hemostasis after a single rpFVIII dose, including one with a baseline FVIII inhibitor titer of 105.8 BU/mL. However, some patients with high‐titer inhibitors may have antibodies that cross‐react with porcine FVIII in vitro [7]. In such patients, rpFVIII may not provide sufficient initial hemostasis. This could make the sequential approach difficult to apply, and the optimal management of such cases remains to be established.

In addition, a previous study reported that repeated rpFVIII exposure induced anti‐porcine antibodies and reduced effectiveness [3]; therefore, minimizing rpFVIII exposure is desirable. Pre‐emicizumab cohort data further indicate that first‐line rpFVIII required less total product than salvage treatment after bypassing agents [8]. These findings align with the sequential strategy of using rpFVIII for rapid hemostasis followed by early emicizumab, and our case series provides preliminary evidence of its feasibility and effectiveness while limiting repeated rpFVIII exposure.

The present study is limited by its small sample size and the absence of anti‐porcine antibody assays. We also did not compare this approach with other hemostatic agents, such as rFVIIa or aPCC; therefore, relative efficacy and safety remain unclear. These factors need to be addressed in future prospective studies.

## 5 Conclusion

In three adults with AHA, a single dose of rpFVIII for acute hemostasis followed by early emicizumab and concurrent immunosuppression achieved rapid bleeding control and sustained prophylaxis without breakthrough bleeding or thrombosis. Prospective studies are needed to compare this strategy with other hemostatic agents, define optimal timing and dosing, and clarify long‐term outcomes.

## Author Contributions

**Yusuke Yamada**: investigation, writing – original draft. **Takashi Onaka**: conceptualization, investigation, supervision. **Kazunori Imada**: supervision.

## Funding

The authors have nothing to report.

## Conflicts of Interest

Takashi Onaka and Kazunori Imada received honoraria from Takeda Pharmaceutical Co. Ltd and Chugai Pharmaceutical Co. Ltd. The other author declares no conflicts of interest.

## Data Availability

Data is available upon reasonable request.
